# A comparative analysis of the response of the hepatic transcriptome to dietary docosahexaenoic acid in Atlantic salmon (*Salmo salar*) post-smolts

**DOI:** 10.1186/s12864-015-1810-z

**Published:** 2015-09-07

**Authors:** Brett D. Glencross, Christian De Santis, Beatrix Bicskei, John B. Taggart, James E. Bron, Monica B. Betancor, Douglas R. Tocher

**Affiliations:** CSIRO Aquaculture, GPO Box 2583, Brisbane, QLD 4001 Australia; Institute of Aquaculture, School of Natural Sciences, Stirling University, Stirling, FK9 4LA Scotland, UK

**Keywords:** Essential fatty acid, DHA, Gene expression, Transcriptome, Atlantic salmon, Microarray

## Abstract

**Background:**

The present study aimed to explore the impact of dietary docosahexaenoic acid (DHA) on aspects of the metabolism of Atlantic salmon (*Salmo salar*). The effects of diets containing increasing levels of DHA (1 g kg^−1^, 3 g kg^−1^, 6 g kg^−1^, 10 g kg^−1^ and 13 g kg^−1^) on the liver transcriptome of post-smolt salmon was examined to elucidate patterns of gene expression and responses of specific metabolic pathways. Total RNA was isolated from the liver of individual fish and analyzed using a custom gene expression 44K feature Atlantic salmon oligo-microarray.

**Results:**

The expression of up to 911 unique annotated genes was significantly affected by dietary DHA inclusion relative to a low DHA reference diet. Analysis of a total of 797 unique genes were found with a significant linear correlation between expression level and dietary DHA. Gene-Set Enrichment Analysis (GSEA) identified a range of pathways that were significantly affected by dietary DHA content.

**Conclusions:**

Pathways that showed a significant response to dietary DHA level included those for long-chain polyunsaturated fatty acid biosynthesis, fatty acid elongation, steroid biosynthesis, glycan biosynthesis, protein export and protein processing in the endoplasmic reticulum. These findings suggest that in addition to clear roles in influencing lipid metabolic pathways, DHA might also have key functional roles in other pathways distinct from lipid metabolism.

## Background

Most aquatic species have some requirement for essential fatty acids (EFA) as dietary nutrients (reviewed by Glencross [1]). However, the specific class of fatty acids (n-3 or n-6, long-chain (>C_20_ or C_18_), their concentrations, and interactions within the diet, appear to vary among species. It is generally considered that marine species have a higher or more defined requirement for the long-chain polyunsaturated fatty acids (LC-PUFA), such as docosahexaenoic acid (DHA; 22:6n-3) or eicosapentaenoic acid (EPA, 20:5n-3), while diadromous species have a reduced requirement and some freshwater species appear to have no requirement at all [2–4]).

Studies examining the dietary requirement for EFA in salmonids are amongst the earliest performed in fish species. The first comprehensive series of studies on EFA requirements of a salmonid were those of Castell et al. [5] with rainbow trout (*Oncorhynchus mykiss*). These authors identified a dietary requirement of trout for α-linolenic acid (LNA, 18:3n-3), however it has since become recognised that the value of LNA as an EFA for trout and Atlantic salmon exists only because these species have the ability to desaturate and elongate LNA to more biologically active fatty acids such as EPA and DHA [3, 6, 7]. Further studies have also shown that both EPA and DHA exert distinct effects on metabolism of these species, but that DHA appears more biologically active as an EFA [8–10].

Based on recent work with post-smolt Atlantic salmon (*Salmo salar*) examining the response to varying levels of dietary DHA, a requirement for around 10 g kg^−1^ was identified [10] ). Post-smolt Atlantic salmon were used as they are the first stage of the marine life-phase of this species, a stage which is the largest user of feed and EFA in commercial aquaculture. However, a range of other biochemical and metabolic effects of dietary DHA were also noted [9] ). While it was evident from the previous study that the pathways of fatty acid elongation and desaturation involved in the biosynthesis of LC-PUFA themselves were affected at a transcriptional level by dietary DHA inclusion level, other studies examining the effects of nutritional variables on broader physiological and molecular responses in fish have shown that there are often a suite of metabolic pathways affected by diet compositions [11–15]. These accessory pathways are also important, possibly key, and provide a broader perspective on the extent that nutritional variables can have on biological functions and those loci at which they act [16]. While at a phenomic level a simple growth bioassay remains arguably the most appropriate and practical way to examine the effects of many nutritional variables, the use of molecular and genomic tools is becoming increasingly important in detecting physiological and biochemical mechanisms not otherwise evident at macroscopic level [17]. Of the expanding genomic technologies available, microarray provides established methodology proven to provide broad utility in examining effects beyond the primary focus [13, 18].

In the present study we aimed to determine the impact of dietary DHA on the metabolism of Atlantic salmon by determining the effects on gene expression in the liver, given that this organ is the primary one involved in LC-PUFA synthesis [3]. To this end, we investigated the response of the hepatic transcriptome of post-smolts fed graded levels of DHA as part of a recently published growth trial [10]. We hypothesized that, in addition to the expected responses of specific genes in the LC-PUFA biosynthesis pathways (e.g. *FADS2, ELOVL5*) that were described recently [9], dietary DHA would also impact other genes and metabolic pathways key to the metabolic response to this important nutrient and thus further elucidate critical functional roles. The combination of a series of graded levels of dietary DHA and the use of a well-established, customized, 44K microarray would facilitate the identification of genes and pathways influenced by varying DHA level. Thus, the study design enabled us to use a regression analysis approach of DHA dose against expression of each gene that would enable identification of responses otherwise insensitive to normal pair-wise molecular comparisons, but potentially highly biologically relevant. Coupling these analytical tools to the expression of both specific genes and the cumulative effect of these genes on pathways, we aimed to elucidate effects of dietary DHA content on fish physiology and, in doing so, provide further insight into the functional roles of this important EFA in fish.

## Methods

### Background experiment

A single basal diet, similar to commercial diets, but marginally lower in lipid to force the composition of the lipid as a point of sensitivity, was formulated and prepared to provide 460 g kg^−1^ protein and 200 g kg^−1^ of lipid at a gross energy level of 22.0 MJ kg^−1^ (estimated digestible protein (DP) and energy (DE) of 440 g kg^−1^ and 19.5 MJ kg^−1^, respectively, with a DP:DE of 22.5 g/MJ). Of the 200 g kg^−1^ of lipid in the diets 185 g kg^−1^ was vacuum infused post-extrusion and it was at this point that the various dietary treatments were produced by using different oil blends. Details on the production of the extruded pellet diets are given in Glencross et al. [10]. Essentially, a series of five dietary DHA inclusion levels were created by blending a range of oils, including an algal derived (*Crypthecodinium* sp.) DHA oil source (DHASCO) combined with the use of butter fat and olive oil. The detailed diet formulations and compositional analysis are provided in Table 1.

**Table 1:** Formulations and compositions of the experimental diets (all values are g kg^−1^). Derived from Glencross et al. (2014) [10]

	D1	D5	D10	D15	D20
*Raw materials used*					
Defatted fishmeal	300.0	300.0	300.0	300.0	300.0
Soy protein isolate	221.0	221.0	221.0	221.0	221.0
Wheat flour	155.0	155.0	155.0	155.0	155.0
Pregelled starch	60.0	60.0	60.0	60.0	60.0
Wheat gluten	60.0	60.0	60.0	60.0	60.0
CaPO_4_	5.0	5.0	5.0	5.0	5.0
Vitamin/Minerals	5.0	5.0	5.0	5.0	5.0
DL-Methionine	4.0	4.0	4.0	4.0	4.0
L-Histidine	1.5	1.5	1.5	1.5	1.5
L-Lysine	1.5	1.5	1.5	1.5	1.5
Yttrium oxide	1.0	1.0	1.0	1.0	1.0
L-Threonine	1.0	1.0	1.0	1.0	1.0
Olive oil	92.5	88.3	82.0	77.8	71.5
Butter fat	92.5	88.3	82.0	77.8	71.5
DHASCO	0.0	8.4	21.0	29.4	42.0
*Composition as measured (g kg* ^*−1*^ *)*					
Dry matter	958	967	952	961	943
Protein	525	526	511	513	521
Fat	181	176	204	205	204
Carbohydrate	212	226	217	213	204
Ash	82	72	68	69	71
Gross Energy	22.3	22.4	23.1	22.7	22.1
Protein:Energy (g MJ^−1^)	23.5	23.5	22.1	22.6	23.6
Total Saturates	56	51	62	65	67
Total Monounsaturates	86	81	89	86	80
Total PUFA	10	12	12	10	10
Total LC-PUFA	2	5	10	14	17
Total n-3	2	6	9	12	15
Total n-6	9	12	13	12	12
DHA	1	3	6	10	13

Twenty Atlantic salmon post-smolts of initial weight 110.9 ± 2.61 g (mean ± S.D.) were allocated to each of 15 × 500 L tanks. The experiment was conducted using a flow-through seawater system at ambient temperatures (14.0 ± 0.82 °C; mean ± S.D.) and dissolved oxygen content (7.8 ± 0.60 mg L^−1^; mean ± S.D.) for a duration of 62-days. Three replicates (tanks) were used for each treatment. A summary of the fish performance data obtained from the growth experiment is presented in Table 2. During the feeding trial an outbreak of amoebic gill disease occurred and the fish were effectively treated by freshwater bathing. There were no treatment biases in the incidence of the disease or its presence at the end of the study [10]. At the end of the feeding trial, all fish within each tank were anesthetized using benzocaine. After each fish was individually weighed, four fish from each tank were euthanized and the apical tip of each liver collected and placed within a 1.5 mL Cryotube™ (*n* = 12 per dietary treatment) and immediately frozen in liquid N_2_ before being stored at -80 °C prior to analysis.

**Table 2:** Growth parameters and feed utilisation. Shown are the regression statistics. Derived from Glencross et al. (2014) [10]

	D1	D5	D10	D15	D20	R^2^	F-value	*P*-value
Initial weight (g/fish)	110.8	112.5	110.7	113.7	109.2	0.004	0.058	0.813
Final weight (g/fish)	226.8	226.7	233.1	232.1	231.4	0.106	1.54	0.236
Weight gain (g/fish)	116.0	114.2	122.5	118.5	122.1	0.112	1.64	0.222
Gain rate d0-d62 (g/d)	1.87	1.84	1.98	1.91	1.97	0.112	1.64	0.222
Gain rate d42-d62 (g/d)	2.30	2.66	2.80	2.70	2.90	0.250	4.34	0.058
Feed intake (g/fish)	106.3	105.9	108.5	105.3	107.3	0.054	0.747	0.403
Feed Conversion (feed : gain)	0.95	0.96	0.90	0.90	0.90	0.224	3.762	0.074

### Transcriptomic analysis

Transcriptomic analysis was conducted using a custom-designed 4 × 44K Atlantic salmon oligo microarray (Agilent Technologies, Wokingham, UK; ArrayExpress accession no. A-MEXP-2065). The salmon microarray and laboratory procedures utilized in this study have been widely used and validated in many previous studies (e.g. Martinez-Rubio et al. [15, 19]; Morais et al. [20, 21]; Tacchi et al. [22]; Bicskei et al. [23]). Briefly, total RNA was extracted from individual samples using TRI Reagent according to manufacturer instructions (Sigma-Aldrich, Dorset, UK), including a high salt precipitation as recommended for polysaccharide-rich tissues such as liver [24]. RNA quantity, integrity and purity were assessed by agarose gel electrophoresis and spectrophotometry (NanoDrop ND-1000, Thermo Scientific, Wilmington, USA). Equal amounts of RNA from two individual fish livers from the same tank were pooled together and analyzed as a single biological replicate, providing two replicates per tank and six replicates per dietary treatment. The resulting RNA samples were amplified using TargetAmp™ 1-Round Aminoallyl-aRNA Amplification Kit, (Epicentre Technologies Corporation, Madison, Wisconsin, USA) following recommended procedures and purified with a RNeasy Mini Kit (Qiagen, Manchester, UK). Aminoallyl-amplified RNA (aRNA) samples were labelled with Cy3 dye (GE HealthCare Life Sciences, Buckinghamshire, UK) while a pool of all aRNA samples was labelled with Cy5 dye (GE HealthCare Life Sciences) and used as a common reference. Unincorporated dye was removed by purifying the labeled aRNA samples with Illustra AutoSeq G-50 dye terminator columns (GE HealthCare Life Sciences). Successful dye incorporation and sample integrity was assessed for 1 μL aliquots of labeled samples by agarose gel electrophoresis followed by fluorescent detection of aRNA products (Typhoon scanner, GE Healthcare Life Sciences). Cy dye concentration and aRNA quantification was measured by Nanodrop mediated spectrophometry.

Labelled aRNA samples were hybridized to the custom-designed array. A dual-label common reference design was adopted where equal amounts (825 ng) of each individual aRNA sample and the common reference pool were competitively hybridized to one array. Samples were processed with the Gene Expression Hybridization Kit (Agilent Technologies), applied to the arrays and immediately incubated using SureHyb hybridization chambers in a DNA Microarray Hybridization Oven (Agilent Technologies) at 65 °C for 17 h. Throughout the experiment, sample processing and hybridization was structured to avoid samples from the same treatment being overrepresented in a particular batch in order to prevent unintentional biases. Scanning was performed using a GenePix 4200 AL Scanner (Molecular Devices (UK) Lid. Wokingham, UK) and the resulting images analyzed with Agilent Feature Extraction Software v.9.5 (Agilent Technologies) to extract the intensity values and identify the features. The foreground intensity was computed as the mean value of pixels, considered a better estimator as being less susceptible to distortion from outlier values [25], while background intensities were computed as the median value of pixels.

### Data pre-processing and differentially expressed genes discovery

Transcriptomic data analysis was performed using R v.3.0.1 and Bioconductor v.2.13 [26, 27]. Quality control, data pre-processing and identification of differentially expressed features/genes were conducted using the package Limma [28]. Features considered outliers in more than 30 % of the replicates within at least one treatment were excluded from further analyses. Foreground intensities were background-corrected using the *normexp* approach (maximum likelihood variant “mle”, offset = 50) as previously reported as the most reliable method for two-colour microarrays where background estimates are available (Ritchie et al. [29]; Silver et al. [30]). Data were log-transformed and normalized using the function *normalizeWithinArrays* (method = “loess”). A second normalization was performed using the *normalizeBetweenArrays* function (method = “RQuantile”) [31]. Filtering of control and features expressed just above background were also removed.

Features of the array were annotated using BLAST 2.2.29+ (blastx) against the entire non-redundant protein database as well as using the KEGG Automatic Annotation Server to obtain functional annotations [32]. A total of 92.8 % of all probes were returned with a BLAST annotation with e-value < 10 (89.6 % with e-value < 0.001), while 59 % of probes were returned with a functional annotation using the KAAS server. Differentially expressed features between treatments were estimated by least squares fitting of linear models on a probe-by-probe basis using the entire pre-processed dataset. The function *lmFit* was used to compute differential expression and statistics were extracted using *ebayes* (trend = TRUE) both functions of the package limma [28]. Contrasts with *p* < 0.05 were considered significant (with no multiple correction adjustment). Genes showing correlation between expression levels and dietary DHA content were identified by linear regression fit using limma with a cutoff of *p* = 0.05. Features representing the same target gene as implied from KEGG annotation were merged into a unique value obtained by selecting the feature with the highest F-value. This allowed a new dataset to be produced where each gene was only represented by one feature in order to avoid redundancy. Merging features resulted in a dataset of 6434 annotated features targeting unique genes.

### Gene-Set Enrichment Analysis (GSEA)

Unique annotated sequences were analyzed using the function *gage* of the package GAGE (Generally Applicable Gene-set Enrichment [33]) to identify mechanistic changes as suggested by coordinated expression changes in gene-sets. Only pathways where genes were perturbed in the same direction (same.dir = T) are reported in this study in order to define the trend of direction change of the underlying genes, however two-direction pathways (same.dir = F) were also analyzed to confirm the presence of other potentially important trends. Differentially expressed gene-sets were determined by pairwise comparison using D1 as a reference treatment. Gene-sets with *q*-values < 0.1 were considered significant, where the *q*-value represented the *p*-value adjusted for false discovery rate.

## Results and discussion

The present study, following on from the phenomic data presented in Glencross et al. [10] ), showed that the incremental inclusion of DHA in the diet of post-smolt Atlantic salmon caused a range of responses in the hepatic transcriptome. These responses were separated into different pathways/biochemical processes to aid interpretation and discussion of their potential functional effects and biological relevance.

### Transcriptomic analysis overview

Overall, analyses revealed that RNA extracted from the liver of fish across the treatment series (D5, D10, D15 and D20) showed between 773, 631, 911 and 786 genes respectively that were differentially expressed (DE) relative to that of the reference treatment (D1) when examined on a pair-wise basis. This differential expression varied subject to treatment comparison, but in each case this was around 10 – 14 % of the total genes available on the microarray for analysis. There were an increasing number of DE genes from livers of fish fed the higher levels of DHA relative to treatment D1. A total of 797 unique and annotated genes were identified using a linear regression model applied to the DE log fold-change data against DHA concentration, showing a significant fit to the model (*p* < 0.05). Of these, 454 (57 %) were down regulated and 343 (43 %) were upregulated with increasing DHA dose relative to the D1 reference treatment.

### Gene-set enrichment analyses

**Fig. 1:**
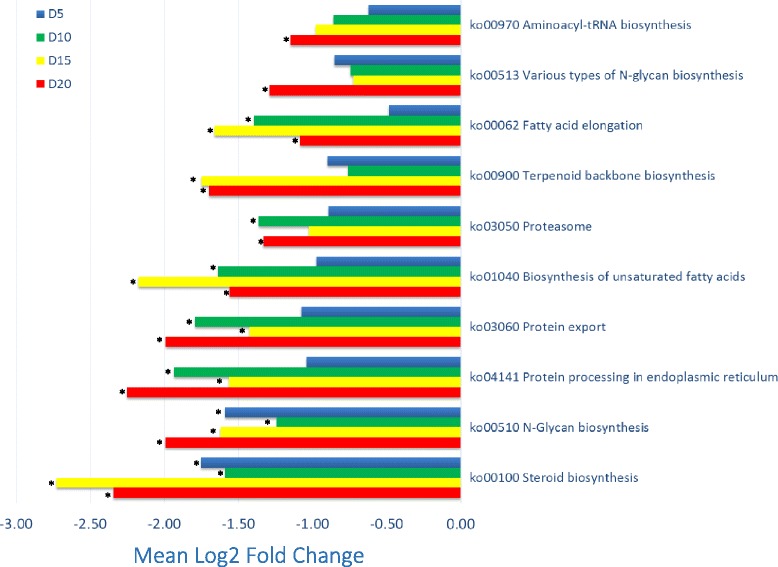
Mean fold change of genes relative to the D1 reference within the pathway responses identified using the GSEA that had at least one significant contrast. Notable is that in each case the mean fold change for each contrast for each pathway was negative. Those contrast q-values that were significantly different to the D1 reference are indicated (*)

The GSEA showed significant enrichment in a range of pathways related to unsaturated (LC-PUFA) fatty acid biosynthesis (ko01040), fatty acid elongation (ko00062), steroid biosynthesis (ko00100), N-glycan biosynthesis (ko00510 and ko00513), terpenoid backbone biosynthesis (ko00900), proteasome (ko03050), protein export (ko03060) and protein processing (ko04141) (Fig. 1). Pathways with significant *q*-values were all notable in that there was a consistent down regulation of most genes in each pathway and the coordinated expression changes in each gene-set were also all cumulatively negatively regulated by dietary DHA. The number of significant treatment contrasts in each of the pathways mentioned ranged from one to all four possible contrasts.

Of those pair-wise contrasts between treatment D1 and each of the other dietary treatments, those that were consistently significantly different for all four comparisons were steroid biosynthesis and N-glycan biosynthesis (ko00510) pathways. Three of the four pair-wise comparisons were significant in the pathways pertaining to unsaturated (LC-PUFA) fatty acid biosynthesis, fatty acid elongation, protein export and protein processing in the endoplasmic reticulum. There were only two comparisons significant for both the terpenoid backbone biosynthesis (ko00900) and proteosome (ko03050) pathways.

There was a broader range of pathways that had indicative trends (q-value < 0.3) based on GSEA. When summarised based on pathway classes, it was noted that pathways in lipid metabolism, the metabolism of terpenoids and polyketides, folding, sorting and degradation, and glycan biosynthesis and metabolism were the most significant (q-value < 0.1) and these were all under-expressed relative to dietary treatment D1. However, of those less significant (0.1 < q < 0.3) notable were the pathway classes of folding, sorting and degradation (down-regulated), signalling molecules and interaction (upregulated), xenobiotics biodegradation and metabolism (upregulated), metabolism of cofactors and vitamins (upregulated), cell communication (upregulated) and the immune system (upregulated).

### Expression of lipid metabolism genes

**Fig. 2:**
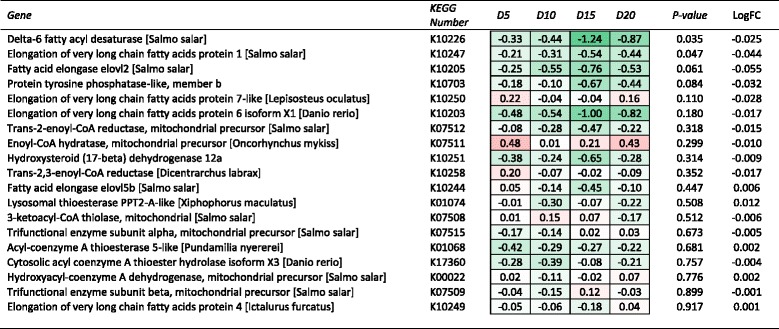
Heat map summary of hepatic expression of fatty acid elongation (ko00062) genes by fish from each DHA inclusion level treatment and their expression (log_2_ fold change) relative to the DHA deficient (D1) treatment. Red and green shading represents the minimal (Min) and maximal (Max) expression levels, respectively. Expression of each gene was also analysed by linear regression against DHA inclusion level. Genes are ranked by regression *P*-value, while mean log_2_ fold change (LogFC) for each gene is also shown

**Fig. 3:**
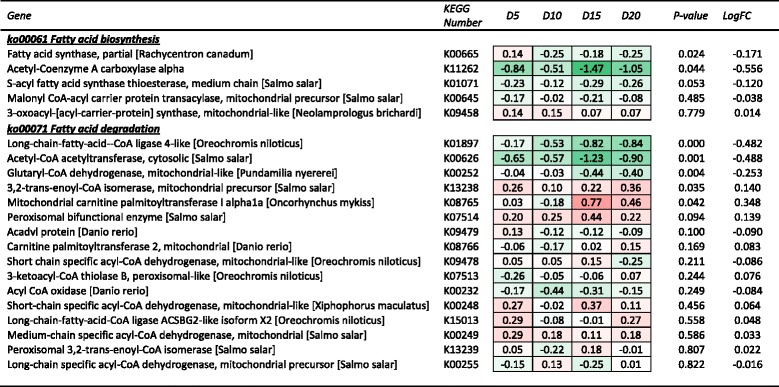
Heat map summary of hepatic expression of fatty acid biosynthesis (ko00061) and degradation (ko00071) genes by fish from each DHA inclusion level treatment and their expression (log_2_ fold change) relative to the DHA deficient (D1) treatment. Red and green shading represents the minimal (Min) and maximal (Max) expression levels, respectively. Expression of each gene was also analysed by linear regression against DHA inclusion level. Genes are ranked by regression *P*-value, while mean log_2_ fold change (LogFC) for each gene is also shown

**Fig. 4:**
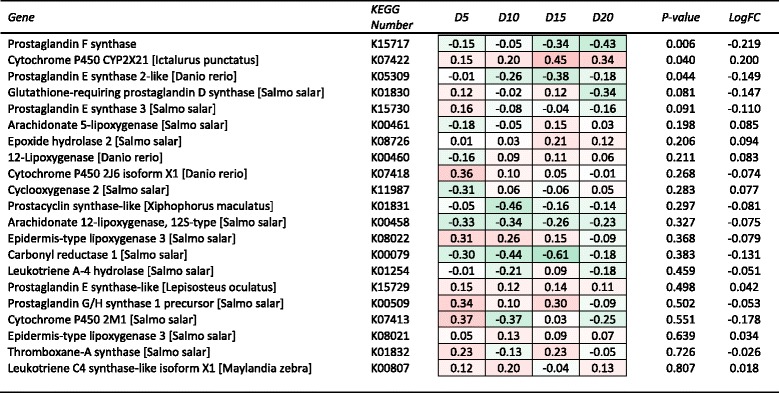
Heat map summary of hepatic expression of arachidonic acid metabolism (ko00590) genes by fish from each DHA inclusion level treatment and their expression (log_2_ fold change) relative to the DHA deficient (D1) treatment. Red and green shading represents the minimal (Min) and maximal (Max) expression levels, respectively. Expression of each gene was also analysed by linear regression against DHA inclusion level. Genes are ranked by regression *P*-value, while mean log_2_ fold change (LogFC) for each gene is also shown

**Fig. 5:**
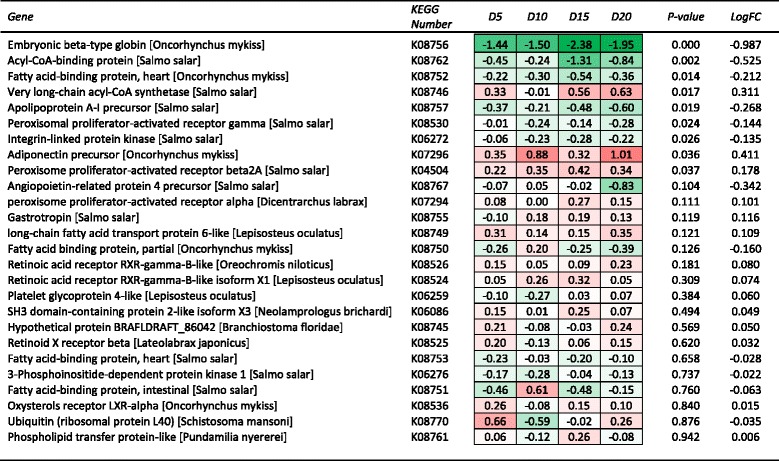
Heat map summary of hepatic expression of PPAR signalling pathway genes by fish from each DHA inclusion level treatment and their expression (log_2_ fold change) relative to the DHA deficient (D1) treatment. Red and green shading represents the minimal (Min) and maximal (Max) expression levels, respectively. Expression of each gene was also analysed by linear regression against DHA inclusion level. Genes are ranked by regression *P*-value, while mean log_2_ fold change (LogFC) for each gene is also shown

A series of different approaches to the bioinformatic analysis of the transcriptomic data were undertaken in this study. Initially contrasts were made amongst the treatments for those unique genes within the microarray with a specific focus on those pathways known to be associated with DHA influence. Notably these genes were primarily associated with lipid metabolism including fatty acid elongation (Fig. 2), fatty acid biosynthesis and fatty acid degradation (Fig. 3), genes of arachidonic acid metabolism (Fig. 4) and PPAR signalling pathways (Fig. 5). Within the 6434 unique annotated sequences present on the custom array, 197 genes were identified as being involved in lipid metabolism (KEGG Orthology), and subject to the specific diet treatment between 22 and 48 of these genes were significantly DE, relative to the D1 reference. Of the DE genes, the majority (~70 %) were down regulated relative to the D1 reference. The proportion of genes significantly down regulated was higher in fish fed treatments D10 and D15 (i.e. 86 and 77 %, respectively) than that observed in fish fed treatments D5 and D20 (i.e. 54 and 66 %, respectively). The use of regression analysis against DHA concentration identified 28 genes (14 %) within the lipid metabolism subset with a significant fit. Of these relationships, 17 (61 %) were down regulated with increasing DHA dose and 11 (39 %) were upregulated.

Of the unique annotated sequences, there were five genes associated with fatty acid biosynthesis, 19 with fatty acid elongation and 16 with fatty acid degradation. Key DE genes among the fatty acid biosynthesis and elongation pathways, that were also significantly responsive to dietary DHA level based on regression analysis, included *ELOVL1* (Elongation of very long chain fatty acids protein 1, K10247), *ELOVL2* (Elongation of very long chain fatty acids protein 2, K10205) and *FADS2* (fatty acid desaturase 2 or delta-6 desaturase, K10226) (Fig. 2). Each of these genes was significantly down regulated relative to the D1 reference diet. In contrast, among the fatty acid degradation pathway genes a significant relationship was observed between DHA level and long-chain fatty acid-CoA ligase (K01897) and glutaryl-CoA dehydrogenase (K00252). In contrast to the biosynthesis and elongation pathways, substantially more genes in the fatty acid degradation pathway were upregulated, albeit none of them dramatically so (Fig. 3).

The role of DHA and the other LC-PUFA in regulating lipid metabolism has been the topic of several reviews [1, 2] ). More recently the focus of this work has shifted towards studying the effects at a transcriptional level [9, 17] ). In the present study, the manipulation of dietary DHA content had limited effect on the primary fatty acid synthesis pathways as defined by expression of genes such as acetyl-coenzyme A carboxylase (K11262), fatty acid synthase (K01071) and malonyl CoA carrier protein transacylase (K00645), none of which were significantly responsive to dietary DHA inclusion level.

The inclusion of DHA did however have a clear effect on the expression of genes involved in LC-PUFA biosynthesis, as evidenced by the down regulation of *ELOVL1*, *ELOVL2* and *FADS2* in fish fed increasing levels of DHA relative to the D1 reference diet. This “down regulation” could also be interpreted as upregulated expression in fish fed the D1 treatment relative to those fish fed the higher DHA treatments. This observation was consistent with that reported by Thomassen et al. [8], who also identified that DHA inclusion in the diet had an inhibitory effect on both *FADS2* and *ELOVL2*, but that the inclusion of dietary EPA did not illicit the same response. Irrespective, the functional effect in the present study is similar, fish fed higher levels of DHA expressed lower levels of these biosynthetic genes suggesting decreased endogenous synthesis of DHA [6, 7].

Ostbye et al. [34] observed increased expression of the β-oxidation linked genes acyl-CoA oxidase (K00232) and carnitine palmitoyltransferase (K08766) with increased levels of dietary LC-PUFA and, in particular, the addition of high levels of DHA to the diet of Atlantic salmon. However, in the present study, direct correlations between dietary DHA level and the expression of these genes was weak (*P* > 0.1) and, even on a pair-wise basis between D1 and D20, there was only a nominal difference in expression (K00232: –0.15; K08766: 0.15) suggesting that the direct effects of DHA on β-oxidation in the present study were limited. However, Ostbye et al. [34] also noted in their study that the dietary levels of EPA and DHA used were extreme and that this led to peroxidative damage of the mitochondrial membranes in their samples. As such, differentiating such peroxidative effects of high dietary LC-PUFA from functional effects of DHA becomes more difficult to compare with the present study where levels of DHA were not so extreme.

It was suggested by Zheng et al. [35] that endogenous LC-PUFA biosynthesis was regulated by nutritional factors that modulated primarily the expression of fatty acyl desaturases, but not the elongases. The present study provides contrasting evidence to this in that there was an equally strong transcriptional responses in both ELOVL and FADS genes to increasing dietary levels of DHA. Similar observations were reported in the earlier paper from this study, which measured gene expression by RTqPCR, where significant pair-wise differences were observed in a range of LC-PUFA biosynthesis genes between fish fed the D1 and D20 treatments, with significant down regulation of most genes in fish fed the higher DHA level [9]. Martinez-Rubio et al. [14] also reported that there were statistically significant effects of varying dietary lipid levels in Atlantic salmon on the expression of lipid metabolism genes other than those of the fatty acyl desaturases. These authors linked this effect to the absolute dietary intake of LC-PUFA, noting that similar such effects were also observed when studies had varied either the total lipid content of the diet or had replaced LC-PUFA through the use of dietary lipid sources other than fish oils [17, 18, 36].

In terms of fatty acid degradation, down regulation of long-chain-fatty acid-CoA ligase (K01897) with increasing dietary DHA was observed. However, it is of note that this enzyme plays a role, not just in the fatty acid degradation (β-oxidation) pathway, but also in that of fatty acid biosynthesis (ko00061), fatty acid metabolism (ko1212) and PPAR signalling pathway (ko3320), among others. The other key observation from this pool of genes was the relative abundance of genes up-regulated with increasing dietary DHA in contrast to that observed in genes from the fatty acid elongation and biosynthesis pathways. This may reflect decreased expression of β-oxidative capacity when dietary DHA is in short supply and the animal is in a sub-optimal nutritional state and tending to conserve nutrients and energy, resulting in increased efficiency in the utilization of these nutrients as was reported in the earlier phenomic study [10].

### Expression of genes of arachidonic acid metabolism and PPAR signalling pathways

Of the unique annotated sequences, there were 21 genes associated with arachidonic acid metabolism and 26 with the PPAR signalling pathway. The only DE genes among the arachidonic acid metabolism pathway, that were also significantly responsive to dietary DHA level based on the regression analysis, was prostaglandin F synthase (K15717) and Prostaglandin E synthase 2-like (K05309), which were both down-regulated with increasing dietary DHA, and Cytochrome P450 CYP2X21which was up-regulated (Fig. 3). Among the PPAR signalling pathway, there were nine genes that were significantly responsive to diet DHA level based on the regression analysis (Fig. 3).

The nutritional regulation of fatty acyl desaturase gene expression via transcription factors can be influenced both directly (by LC-PUFA acting as ligands) and indirectly (via autocrine hormones such as eicosanoids acting as ligands). The role of LC-PUFA in eicosanoid or arachidonic acid (ARA) metabolism in fish has been recognised for some time [37–39]. In the present study, there was limited impact of variation in dietary DHA levels on the expression of genes in the ARA metabolism pathway (ko00590), with the exception of prostaglandin F and E synthase and a cytochrome P450 (CYP2X21), the first two of which were down-regulated, and the other up-regulated with increasing dietary DHA level. Notably, other key genes in this pathway, such as cyclooxygenase 2 (K11987) and some of the lipoxygenases (K08021, K00460; K00461) were unaffected by dietary DHA. This suggested that a major mode-of-action of DHA on the eicosanoid pathway would be down regulation of the production of prostaglandin F_2_ series autocrine hormones with apparently less impact on other enzymes involved in the production of other eicosanoids, as implicated by the low response by the majority of genes in the pathways to dietary DHA level.

It is generally acknowledged that EPA has a role in acting as a modulator of eicosanoid metabolism by inhibiting the conversion of ARA to other eicosanoids intermediaries via the enzyme COX1 and alternatively itself being converted to eicosanoids with substantially different properties to those produced from ARA metabolism/eicosanoid synthesis pathway [3]. Other studies have also shown a level of suppression of prostaglandin synthesis through the dietary provision of n-3 LC-PUFA [38] ). In the study by Ghioni et al. [38], the provision of 18:4n-3 and 20:4n-3 to a salmonid cell culture inhibited the production of prostaglandin F_2α_ (PGF_2α_) from ARA. In the present study, the down regulation of prostaglandin F synthase (K15717) by increasing dietary DHA was consistent with inhibited production of prostaglandin F, albeit this time demonstrated at a gene transcriptional level. The role of PGF_2α_ in fish is primarily linked to reproductive development, although it has also been implicated in stimulation of the olfactory responses [40]. The suppression of production of PGF_2α_ through the inhibiting action of the n-3 LC-PUFA has also been shown to result in up-regulation of 12-HETE, PGE_1_ and PGF_1α_ production in fish [38].

The effects of peroxisomal proliferator-activated receptors (PPARs) have been linked to fatty acyl desaturase activity, both directly and via a secondary compensatory response attributed to *PPARα* influencing β-oxidation and increasing the overall demand for LC-PUFA [35, 41, 42]. There were few correlations observed between dietary DHA level and the expression of genes in the *PPAR* signalling pathway (ko03320). This is consistent with Ostbye et al. [34], who observed using a RT-qPCR approach an increase in the expression of *PPAR*β with the addition of high levels of DHA to the diet of Atlantic salmon, although the effects observed with increased dietary levels of both DHA and EPA were not as clear. Examination of the correlation between expression of *ELOVL/FADS* genes and *PPAR/FABP* genes in the present study also showed a close relationship (R^2^ values of 0.477 to 0.971), supporting possible linkage between PPARs expression and fatty acid desaturase and elongase expression/activity [42].

In the present study some marginal correlations (q-value < 0.3) were observed for other genes in signalling pathways. Notable pathways such as the *TNF* (ko04668), *NF-kappa B* (ko04064), *PI3K*-*Akt* (ko04151) and the *mTOR* (ko04150) signalling pathways all showed some level of response to dietary DHA and, in nearly all cases, the mean log fold expression was upregulated relative to the D1 reference diet. Similar transcriptional responses of specific genes such as the sterol regulatory element binding protein (*SREBP1*; k07197) and liver X receptor (*LXR*; k08536) to increasing levels of dietary DHA were reported previously [9]. Interestingly, it was demonstrated in mice that expression of the Δ5 and Δ6 *FADS* were both down regulated with over-expression of the transcription factors *SREBP1* and *PPAR*α [43]. This contrasted with the observations in the present study where both these transcription factors and *FADS* appeared to be expressed in parallel.

### Expression of steroid biosynthesis pathway genes

One of the main aims of the present study was to use the large-scale microarray approach to enable the examination of genes and pathways not normally directly associated with the manipulation of dietary LC-PUFA content. Using GSEA we were able to identify a significant negative mean fold change of genes in a suite of pathways, but this was most consistent in the steroid biosynthesis pathway (ko00100). This pathway is primarily involved in the production of steroid hormone precursor, cholesterol, and vitamin intermediaries such as cholecalciferol (vitamin D_3_).

Previous research has shown that manipulation of dietary fatty acid content and composition can influence steroidogenesis in fish [44]. The inhibition of eicosanoid synthesis via interactions between ARA and EPA has been shown to have flow-on effects on steroidogenesis [45]. However, the data presented in the present study showed that the effect of LC-PUFA on steroidogenesis extended beyond the role that ARA and EPA play in influencing eicosanoid metabolism as direct substrates and the subsequent impacts of those autocrine hormones on steroidogenesis. Indeed the range of steroids influenced by these interactions also appeared considerable, with LC-PUFA implicated in affecting cortisol and progesterone among others [46–49].

Increased use of vegetable oils in fish diets, resulting in reduced n-3 LC-PUFA intake, has been reported to alter the post-stress levels of plasma cortisol [47–49]. It was shown that dietary fatty acids can influence the expression of stress response associated genes in some fish species, such as steroidogenesis acute regulatory protein (*StAR*) [49]. The *StAR* regulatory protein has also been reported to be influenced by the down regulation of PGF_2α_ production, which in both the present and previous studies was down regulated in response to dietary DHA itself. Interestingly the influence of this regulatory protein has also been reported to impact on steroid levels independent of transcriptional changes and this suggests that a tandem approach of following both transcription and actual steroid levels would perhaps be appropriate [46]. Notably, in addition to prostaglandin-linked effects, DHA also has the capacity to modulate steroidogenesis via its influence on *PPAR*, which in turn can modulate genes such as *StAR* [50].

Based on the gene expression data, it will be useful to measure levels of circulating steroids (e.g. testosterone, estradiol and cortisone) and steroid precursors (e.g. pregnenolone, progesterone and corticosterone) in future studies as tangible features linking changes in gene expression to physiological effects. This would allow further phenomic validation of the transcriptomic responses observed in the present study.

### Expression of N-glycan biosynthesis pathway genes

In the present study, GSEA also identified a significant impact of DHA in the down-regulation of the N-glycan biosynthesis pathway. This pathway plays an important role in synthesis of glycoproteins and proteoglycans, and these N-linked glycan structures are important in coordinating protein-folding during protein synthesis on the endoplasmic reticulum [51]. Additionally N-linked glycans also play an important role in cell-cell interactions [51]. The identification of effects of dietary DHA on the expression of these genes and other signalling genes provides further support for the complex level of interactions involved in response to n-3 LC-PUFA.

Another form of glycans are the glycosaminoglycans, which include polymers such as keratin, chondroitin and dermatan, each of which play roles as structural carbohydrates in anti-inflammatory responses, coagulation, wound repair and fibrosis in eukaryotic cells [52, 53]. Therefore, the data also imply that this pathway, with important pro-inflammatory processes, is possibly down-regulated by dietary DHA. The identification of differential expression of these genes in response to DHA provides further evidence of the role of LC-PUFA on non-lipid metabolism pathways including inflammatory processes.

### Other pathways influenced by dietary DHA level

Other studies examining variation in dietary n-3 LC-PUFA levels have often reported changes to various immunological parameters [54, 55]. In the present study there were few data to support a systemic impact of DHA on any of the immune system pathways with weak q-values identified from the GSEA for most of the annotated genes/pathways. Of those pathways related to the immune system that were identified it was noted that there was a general upregulation of immune pathways relative to D1 with increasing dietary DHA level. This lends some support to the notion that DHA has effects (albeit possibly indirect) on the immune system, with pathways such as the chemokine signalling (ko04062), FC epsilon RI signalling (ko04664) and Natural Killer cell mediated cytotoxicity (ko04650) pathways all likely influenced by dietary DHA and n-3 LC-PUFA in general. However, the fish were not challenged in this trial and so it is likely that the extent of the impact of DHA on immune pathways was not fully apparent.

A range of pathways were also noted that had little to no activity including amino acid metabolism, cell growth and death, and the endocrine system among others.

## Conclusions

The results of the present study showed that variation in DHA supply in the diet of post-smolt Atlantic salmon resulted in significant changes in the expression of a range of genes, not just those associated with lipid metabolism as might be expected but, indeed, a suite of ‘flow on’ effects in other important pathways. Notably, there was significant enrichment of transcriptional responses observed in other biosynthetic pathways such as steroid (sterol) biosynthesis, glycan biosynthesis, and protein synthesis, export and processing. The effects on steroid biosynthetic pathways in particular provided indication of a potential mechanism of action by which DHA exerts functional effects on a suite of genes through transcriptional changes. The data suggest it would be particularly pertinent in future studies examining the role of DHA (and possibly other LC-PUFA) on the growth of fish, to focus some attention on the levels of plasma steroids to observe which of these signalling molecules is linked to these dietary nutrients, and further our understanding of how DHA and other LC-PUFA elicit their nutritional and physiological impacts.

### Availability of supporting data

The data set supporting the results of this article is available in MIAME-compliant format in the ArrayExpress repository under accession number E-MTAB-3180 (http://www.ebi.ac.uk/arrayexpress/experiments/E-MTAB-3180/).

## Abbreviations

DHA: Docosahexanoic acid
EFA: Essential fatty acids
ELOVL: Elongase of very long-chain fatty acids
FADS: Fatty acid desaturase
GSEA: Gene-Set Enrichment Analysis ()
LC-PUFA: Long-chain polyunsaturated fatty acid
PPAR: Peroxisomal proliferator-activated receptors
RNA: Ribonucleic acid
RT-qPCR: Real-Time Quantitative Reverse Transcription PCR
SD: Standard deviation
